# Availability of Physical Rehabilitation Facilities for People with Disabilities in Iran: A Comparative Study on Universal Health Coverage

**DOI:** 10.34172/aim.2022.109

**Published:** 2022-10-01

**Authors:** Marzieh Shirazikhah, Payam Roshanfekr, Amirhossein Takian, Mehdi Alizadeh Zarei, Adeleh Shirazikhah, Mohammad Taghi Joghataei

**Affiliations:** ^1^Social Determinants of Health Research Center, University of Social Welfare and Rehabilitation Sciences, Tehran, Iran; ^2^Social Welfare Management Research Center, University of Social Welfare and Rehabilitation Sciences, Tehran, Iran; ^3^Director, Department of Global Health & Public Policy, Tehran University of Medical Sciences, Tehran, Iran; ^4^Vice Dean, School of Public Health, Tehran University of Medical Sciences, Tehran, Iran; ^5^Chief of Research, Health Equity Research Centre (HERC), Tehran University of Medical Sciences, Tehran, Iran; ^6^Department of Occupational Therapy, School of Rehabilitation Sciences, Iran University of Medical Sciences, Tehran, Iran; ^7^Office of the Minister’s Adviser on Rehabilitation, Ministry of Health and Medical Education, Tehran, Iran; ^8^School of Advanced Technologies in Medicine, Iran University of Medical Sciences, Tehran, Iran

**Keywords:** Availability, Facilities, People with physical disabilities, Rehabilitation

## Abstract

**Background:**

Iran, like many other countries, has committed to providing universal and equal access to health care and rehabilitation for people with disabilities by joining the Convention on the Rights of People with Disabilities. Thus, this study aimed to examine the availability of rehabilitation facilities on national and sub-national levels.

**Methods:**

This cross-sectional study was conducted between May and December 2019. The data of rehabilitation facilities including infrastructure and rehabilitation workforce in health system settings were obtained using rehabilitation master list. The data were collected from the Vice-Chancellor for the Treatment Ministry of Health and Medical Education, the Rehabilitation of the State Welfare Organization, and Licensing and Planning the Medical Council in the 32 provinces of Iran and analyzed using Excel version 2016.

**Results:**

On the national level, the following situation was found: in inpatient settings: 1.1 beds per million population; in outpatient settings: physiotherapy 42.6, optometry 16.4, audiology 10.5,occupational therapy 8.2, speech therapy 8.1, orthotic & prosthetic 4.5, physical medicine & rehabilitation 3.8 centers; day-care centers 11.7 and rehabilitation centers 1.3 centers, community-based rehabilitation (CBR): 15.9 units, rehabilitation home care 2 centers, rehabilitation nursing home care 1.6 centers and medical rehabilitation home care 0.3; Long-term care centers: residential care 4.1 centers per million population. Regarding rehabilitation work force: physiotherapists 84, speech therapists 34.8, occupational therapists 32.5, optometrists 31.2, audiologists 27.9, prosthetists and orthotists 10.3 therapists and physical medicine & rehabilitation 5.1 specialists per million populations. On the sub-national level, there were no outpatient rehabilitation centers in 12 of the provinces and the distribution of day rehabilitation centers in the rich provinces was 10 times higher. The number of CBR units, rehabilitation home care and rehabilitation nursing home were 40, 22, and 23 times higher in rich provinces than in poor provinces, respectively and there were no medical rehabilitation home care centers in 21 provinces. Regarding long-term care, the residential care centers in the richest province were 8 times higher.

**Conclusion:**

According to the WHO report and the rehabilitation expert panel, it was concluded that the number of rehabilitation facilities including rehabilitation centers and workforce was limited in Iran and that the available centers were also poorly distributed in the provinces of the country. This made it difficult for people to have fair access to rehabilitation services. It appears that health policymakers should pay special attention to further developing rehabilitation facilities.

## Introduction

 “Universal health coverage means that all people have access to the health services they need, when and where they need them, without financial hardship. It includes the full range of essential health services, from health promotion to prevention, treatment, rehabilitation, and palliative care”.^1^ In Iran, as in other countries, with the increase in the elderly population and the number of chronic diseases, the prevalence of disability has increased and thus the need for achieving universal health coverage has increased more than before.^2,3^ Rehabilitation services are not limited to “people with disabilities”, and all members of the community can benefit from them.^4^ In the absence of rehabilitation services and lack of access to them, the consequences of diseases or injuries increase in individuals and limit their activities and participation in the society. This ultimately reduces the quality of life and increases treatment and rehabilitation costs for people with disabilities.^1,5,6^ One of the prerequisites for achieving universal health coverage is the availability of appropriate services.^7^ Regular monitoring of the availability and distribution of services is often a challenge for any country’s health system. Therefore, policymakers and health planners need strong evidence so that they can make decisions about service availability, needs, resource allocation, monitoring, and development.^8^ To achieve this goal, various organizations should be investigated periodically concerning the availability of and accessibility to services on national and sub-national levels. These investigations include obtaining up-to-date and accurate information on the basic components of the availability of facilities, i.e. information about the availability of health care workforce and infrastructure in the public and private sectors.^9^

 So far, various tools have been used to obtain information about the availability of rehabilitation services.^10,11^ One appropriate tool in this regard is the service availability and readiness assessment (SARA) survey that determines the availability of services using a set of indicators.^12,13^ This survey was used in Iran in 2015 to prepare a master list of rehabilitation services for the integrated evaluation of inpatient, outpatient, community-based, and long-term rehabilitation services on the national level.^14^ Accordingly, the present study was designed to obtain information on national and sub-national levels over a long period. The aim was to use a suitable and comprehensive tool to collect various data related to the infrastructure and rehabilitation workforce collected in an integrated manner in all health system settings so that it can provide the required evidence for policymakers, executives, and researchers to develop universal rehabilitation coverage

## Materials and Methods

 This cross-sectional study was conducted between May and December 2019. In this study, the master list of rehabilitation services was used to collect data. The list was developed in the study by Shirazikhah et al to identify the availability of rehabilitation services in Iran.^4^ The SARA survey was used in the study and based on the results of the two focus group discussions, the list of rehabilitation facilities in Iran was obtained. The list has two main sections: 1) rehabilitation infrastructure, and 2) rehabilitation workforce. The availability of rehabilitation infrastructures in different health system settings such as inpatient, outpatient, community-based and long-term settings was determined separately in different provinces of the country in public and private sectors. Moreover, the availability of rehabilitation workforce including physical medicine and rehabilitation specialists, physiotherapists, occupational therapists, speech therapists, audiologists, optometrists, and prosthetists and orthotists was examined in all the provinces.

 The data were collected from the Vice-Chancellor for the Treatment Ministry of Health and Medical Education, the Vice-Chancellor for Rehabilitation of the State Welfare Organization, and the Vice-Chancellor for the Licensing & Planning the Medical Council in the 32 provinces of Iran. Three researchers receiving necessary training in this regard were used to examine the data in terms of accuracy, precision, and internal compatibility.

 The data were analyzed on national and sub-national levels. The quantitative data were reported based on the number per million populations. The data were analyzed using Excel version 2016. We use Microsoft Excel conditional formatting function 3-color method where green to white scale displays three color gradient in a range of cells (maximum-minimum)/4, each color represents a quadrant. The shade of the color represents the value of the cell, with darker standing for higher value. Finally, the results were compared with the reports of the World Health Organization (WHO) and the opinions of rehabilitation specialists in the form of an expert panel.

## Results

 The data related to the infrastructures of rehabilitation services in Iran was obtained concerning inpatient, outpatient, community-based, and long-term rehabilitation in 2018. Accordingly, only two rehabilitation hospitals with 85 beds (1.1 per million population) in Tehran had inpatient services. Infrastructures for outpatient rehabilitation in Iran included rehabilitation, physical medicine and rehabilitation, physiotherapy, occupational therapy, speech therapy, audiology, optometry, orthotic and prosthetic, and daycare centers. There were1.7 rehabilitation centers per million populations in 19 out of the 31 provinces in Iran, with the provinces of Isfahan, Yazd, Golestan, Hamedan, and Tehran having the highest number of these centers, respectively. The remaining provinces did not have any rehabilitation centers and only had an average of 3.8 physical medicine and rehabilitation centers per million populations, with the provinces of Fars, Tehran, Isfahan, East Azarbaijan, and Qom having the highest number of these centers, respectively. Five of the provinces did not have any physical medicine centers (Table 1).

**Table 1:** Number of Rehabilitation Facilities Per 1000000 Population in 2018

**Province**	**Outpatient**									**Community Center Rehabilitation**				**Long-term Care**
	**Rehabilitation Center**	**Office**							**Day care Center**					
		**Physical Medicine & Rehabilitation **	**Physiotherapy **	**Occupational Therapy **	**Speech Therapy **	**Audiometry **	**Orthotic& Prosthetic **	**Optometry **		**Rehabilitation Home Care**	**Medical Rehabilitation Home Care**	**Rehabilitation Nursing Home Care**	**CBR Unit**	**Residential Care**
Total	1.7	3.8	42.6	8.2	8.1	10.5	4.5	16.4	11.7	2	0.3	1.6	15.9	4.1
	(0.0-5.3)	(0.0-7.8)	(15.4-112.2)	(1.1-18.1)	(0.9-18.1)	(4.0-22.4)	(1.4-26.2)	(4.6-33.8)	(5.1-52.7)	(0.6-13.7)	(0.0-1.3)	(0.0-7.1)	(2.5-56.7)	(1.1-9.1)
Alborz	1.8	2.9	40.2	18.1	18.1	12.2	8.1	29.9	7	0.7	0	0	2.6	8.5
Ardabil	0	2.4	26	3.9	3.9	8.7	7.1	10.2	11	0.8	0	7.1	12.6	3.1
Bushehr	0	1.7	33.5	6.9	6	12.9	3.4	14.6	8.6	0.9	0	0	18.5	2.6
Chahar Mahaal & Bakhtiari	0	3.2	40.1	11.6	9.5	7.4	10.6	6.3	46.4	13.7	1.1	0	28.5	3.2
East Azerbaijan	2	5.1	29.9	3.8	3.8	5.4	2.8	4.6	12.8	1.8	0.5	1.3	14.6	2.6
Fars	1.6	7.8	48.9	10.5	9.9	9.5	8.7	21	7.2	1	1	3.1	9.7	2.3
Gilan	2.4	3.2	61.6	7.1	5.1	8.7	4.7	18.6	10.3	3.2	0.4	1.6	14.2	5.9
Golestan	4.3	1.1	46	5.9	7	8	8.6	16.6	22.5	3.2	0	2.7	16.6	1.6
Hamadan	2.9	2.9	25.9	6.9	7.5	8.6	13.8	15.5	6.3	1.7	0	3.5	14.4	1.7
Hormozgān	0	1.7	18.6	4.5	5.1	5.6	1.7	10.1	5.1	1.1	0	0.6	13.5	1.1
Ilam	1.7	0	41.4	6.9	10.3	20.7	10.3	13.8	24.1	10.3	0	1.7	29.3	3.4
Isfahan	5.7	7.2	51.4	8.4	8.8	10	15.8	10.7	13.9	3.7	0.2	1.2	20.5	6.6
Kerman	2.2	1.9	30	6.3	7	7.3	5.4	9.2	10.7	1.9	0	2.2	23.4	1.6
Kermanshah	0.5	1.5	42	6.1	4.6	12.8	7.7	19	12.8	1	0.5	1	14.3	3.1
Khuzestan	0.2	1.5	77.3	5.7	5.3	10.6	4	12.5	14.2	1.3	0	1.3	8.49	1.9
Kohgiluyeh & Boyer Ahmad	0	0	112.2	8.4	4.2	8.4	1.4	9.8	18.2	1.4	0	0	37.9	2.8
Kurdistan	0	1.2	34.3	5.6	6.9	12.5	5.6	12.5	8.1	1.9	0	1.2	11.2	2.5
Lorestan	0	1.7	31.2	4.5	4	9.7	10.2	14.8	18.7	3.4	0	1.7	16.5	2.3
Markazi	1.4	4.2	15.4	15.4	6.3	22.4	9.1	14.7	11.9	0.7	0	4.2	21	4.9
Mazandaran	0.9	3.4	64.6	9.7	12.5	12.2	7.3	18.9	13.4	3	0	2.4	5.8	2.1
North Khorasan	0	0	29	8.1	9.3	12.7	11.6	27.8	18.5	2.3	0	4.6	19.7	7
Qazvin	0	2.4	33	9.4	4.7	8.6	6.3	14.9	12.6	2.4	0	1.6	9.4	5.5
Qom	1.5	4.6	32.5	10.1	16.3	16.3	7	18.6	9.3	1.5	0	0	6.2	2.3
Razavi Khorasan	0.9	1.4	35.4	8.4	9.8	8.9	7.1	33.1	7.3	0.9	0.3	1.7	56.7	7
Semnan	1.4	0	58.4	10	12.8	11.4	2.8	14.2	28.5	4.3	0	0	31.3	5.7
Sistan and Baluchestan	0.4	0.4	16.6	1.1	1.8	4	1.8	13.3	7.2	1.1	0	1.1	14.4	1.1
South Khorasan	0	0	29.9	10.4	11.7	4	6.5	33.8	33.8	9.1	1.3	2.6	23.4	9.1
Tehran	2.7	7.4	50.2	11.3	9.8	13.3	26.2	15.7	5.9	0.7	0.9	1.3	1.4	6
West Azerbaijan	0	4	27.9	4.9	7.4	11.6	6.1	7.4	14.4	0.6	0	0.3	13.2	4.6
Yazd	5.3	3.5	63.2	5.3	4.4	8.8	14.1	17.6	52.7	9.7	0	2.6	10.5	4.4
Zanjan	0	2.8	27.4	6.6	0.9	5.7	5.7	9.5	11.3	1.9	0	0	50.1	1.9

Note: Each color represents a quadrant (range=maximum-minimum)/4. The shade of the color represents the value of the cell, with darker standing for higher value. This means that in each column that represents one of the rehabilitation facilities, the number of those facilities in the province increases from white to dark green.

 There were physiotherapy, optometry, audiology, occupational therapy, speech therapy, orthotic and prosthetic, and daycare centers with an average of 42.6, 16.4, 10.5, 8.2, 8.1, 4.5, and 11.7 per million population, respectively. Regarding community-based rehabilitation (CBR), the country provided rehabilitation home care services with an average of two centers per million population in the all the provinces, medical rehabilitation home care services with an average of 0.3 centers per million population (24 of the provinces had no centers), rehabilitation nursing home care services with an average of 1.6 centers per million population (six of the provinces had no centers), and CBR units with an average of 15.9 units per million population, and long-term care centers with an average of 4.1 centers per million population (Table 1).

 Concerning rehabilitation workforce, there were physiotherapists, speech therapists, occupational therapists, optometrists, audiologists, prosthetists and orthotists, and physical medicine and rehabilitation specialists with an average of 84.1, 34.8, 32.5, 31.2, 27.9, 10.3 and 5.1 people per million populations, respectively. The highest and lowest per capita average was related to physiotherapists (twice as many as the rest of the workforce) and physical medicine and rehabilitation specialists, respectively. In general, the entire workforce grew more than 50% compared to the early 2011, and prosthetists and orthotists grew 22.9 times (Table 2).

**Table 2:** Number of Rehabilitation Workforce per 1000000 Population

**Province**	**Physical medicine & Rehabilitation**		**Physiotherapist**		**Occupational Therapist**		**Speech Therapist**		**Sudiologist**		**Optometrist**		**Prosthetists and Orthotists**	
	**90**	**97**	**90**	**97**	**90**	**97**	**90**	**97**	**90**	**97**	**90**	**97**	**90**	**97**
Total	3.1	5.1	59.2	84.1	15,1	32.5	16.8	34.8	17.3	27.9	24	31.2	0.9	10.3
	(0.0-7.3)	(0.0-10.3)	(12.5-132.3)	(23.6-168.7)	(0.64-48.59)	(3.6-70.7)	(3.5-34.2)	(7.9-67.6)	(0.77-42.4)	(11.3-56.1)	(4.4-50.3)	(6.4-55.8)	(0.0-4.6)	(1.4-26.1)
Alborz	2.44	4.06	59.02	74.84	19.94	31.34	18.32	32.07	21.16	26.91	28.9	42.77	0.81	8.11
Ardabil	4.77	5.51	21.46	31.49	10.33	20.47	4.77	14.96	13.51	24.4	14.31	22.83	0	7.08
Bushehr	2.84	4.3	36.02	39.54	3.79	12.03	10.43	18.91	15.17	22.35	12.32	18.91	0	3.44
Chahar Mahaal & Bakhtiari	0	3.17	39.82	59.09	7.74	70.69	18.81	37.98	9.96	28.49	4.42	12.66	0	10.55
East Azerbaijan	6.66	8.95	33.58	54.99	6.66	24.3	5.86	18.16	9.06	14.58	4.8	6.39	0.27	2.81
Fars	7.32	9.48	85.31	132.75	9.91	32.98	14.65	38.13	10.99	19.79	29.3	42.67	0	8.66
Gilan	0.4	2.37	52	79.82	8.46	18.97	8.87	19.36	11.29	15.81	14.11	21.73	0	4.74
Golestan	0.55	1.07	41.08	68.49	6.19	21.94	8.44	19.26	12.38	18.19	18.57	22.47	0	8.56
Hamadan	2.27	5.18	26.16	36.82	7.96	28.76	10.81	22.44	11.94	28.76	10.81	18.41	0.57	13.81
Hormozgān	0.63	1.13	15.21	23.64	2.53	6.76	3.8	7.88	7.6	11.26	7.6	11.82	0	1.69
Ilam	0	0	33.75	62.05	10.66	37.92	14.21	62.05	12.43	51.71	14.21	18.96	0	10.34
Isfahan	1.02	8.2	58.72	92.17	8.94	34.17	24.38	48.43	13.61	24.8	12.19	15.62	0.61	15.82
Kerman	2.38	3.16	36.07	49.61	9.87	21.49	14.29	38.87	11.91	18.64	16.67	20.85	0.34	5.37
Kermanshah	0	0	31.87	52.75	10.28	21	7.2	21.51	12.85	28.68	21.08	33.29	0	7.68
Khuzestan	0.65	1.27	57.67	83.22	8.49	21.44	14.15	33.12	11.1	26.54	10.66	19.74	0	4.03
Kohgiluyeh & Boyer-Ahmad	0	1.4	37.96	74.33	48.59	61.71	15.18	36.46	18.22	47.68	13.66	23.08	0	1.4
Kurdistan	2.01	3.12	18.75	33.06	4.69	39.3	14.06	34.93	11.38	34.93	10.04	13.63	0	5.61
Lorestan	1.71	3.98	23.37	42.03	2.28	18.74	9.69	27.26	7.41	32.94	5.13	22.39	0	10.22
Markazi	0.71	4.2	37.48	53.87	14.85	35.68	16.97	25.18	19.8	35.68	15.56	28.93	0	9.09
Mazandaran	3.9	4.57	79.05	117.86	11.39	23.45	15.29	36.55	17.89	22.84	24.4	23.08	0	7.31
North Khorasan	0	0	12.53	31.28	6.83	23.17	13.67	27.81	13.67	22.01	31.89	38.23	0	11.59
Qazvin	0	1.57	34.6	52.6	15.65	25.91	9.06	17.27	14	18.06	17.3	25.12	0	6.28
Qom	3.41	6.19	52.9	86.67	20.48	34.82	31.57	50.3	27.3	33.27	28.16	32.5	0	6.96
Razavi Khorasan	1.32	2.33	38.97	67.76	9.04	23.31	14.47	37.77	12.17	17.87	50.31	55.79	0.16	7.15
Semnan	1.56	2.85	73.32	112.48	26.52	44.14	32.76	51.26	18.72	25.63	20.28	28.48	0	2.85
Sistan and Baluchestan	0	0.72	30.05	48.65	1.93	3.6	3.47	14.77	0.77	13.33	26.19	34.95	0	1.8
South Khorasan	1.35	2.6	17.54	40.32	17.54	44.22	21.59	59.83	10.8	20.81	48.58	54.62	0	6.5
Tehran	6.82	10.25	132.35	168.68	43.55	68.81	33.64	54.04	42.41	56.08	43.71	54.87	4.63	26.15
West Azerbaijan	4.81	6.74	25.32	36.75	0.64	16.54	3.53	26.03	5.45	20.52	7.05	13.17	0	6.13
Yazd	0.98	2.63	48.78	71.14	13.66	43.04	34.15	67.63	21.46	32.5	27.32	34.25	0	14.05
Zanjan	2.92	3.78	25.34	34.04	8.77	16.08	7.8	13.24	11.7	17.02	12.67	15.13	0	5.67

Note: Each color represents a quadrant (range=maximum-minimum)/4. The shade of the color represents the value of the cell, with drake standing for higher value. This means that in each column that represents one type of rehabilitation workforce, the number of those workforces in the province increases from white to dark green.

## Discussion

 This study aimed to examine the availability of rehabilitation facilities, including infrastructures and workforce, for people with disabilities using an integrated approach in all settings. Rehabilitation is now offered in different settings in different countries and does not have the same pattern in all countries.^15,16^ Therefore, to investigate the availability of rehabilitation services, all inpatient, outpatient, community-based, and long-term medical rehabilitation services should be informed of short intensive and long-term care.^4^ Based on this study, there is currently approximately one rehabilitation bed per million population in a third-level hospital in the inpatient section.^14^ In second-level hospitals, although medical acute care services, such as stroke care, cerebral trauma care, etc., are provided for patients in different units, such as neurology, orthopedics, and neurosurgery units, rehabilitation services are very limited. In Iran, rehabilitation hospitals are commonly converted into general hospitals.^17^ One major reason for the failure of such hospitals is the lack of insurance coverage for rehabilitation services. This has caused rehabilitation hospitals to use services in general hospitals that are covered by insurance. Moreover, private hospitals are reluctant to set up or maintain inpatient rehabilitation units. However, research shows that rehabilitation services significantly reduce the cost of both rehabilitation interventions and other medical procedures before and after the acute phase.^18^ This is compared with developed countries such as Canada and Australia (with 18 and 24 inpatient rehabilitation beds for every million people).^19^ Reports from European countries have shown that only 29% of countries are able to meet the needs of more than 80% of patients in inpatient rehabilitation centers,^20^ and in some of these countries, rehabilitation is available in the subacute phase that clearly provides a low number of services. Long-term rehabilitation has been lower compared to other rehabilitation settings in Europe.^21^ In a study conducted by Hogan et al, the standard rehabilitation bed was 0.1 per 1000 people.^22^ The British Society of Rehabilitation Medicine has suggested that at least 60 beds per million population should be allocated to rehabilitation specialists in an inpatient unit.^23^ However, this number is smaller in Iran and thus it is necessary to increase the number of rehabilitation beds and provide fair access to rehabilitation services.

 Moreover, the number of outpatient rehabilitation centers and their distribution are structurally inappropriate. In particular, most of the available settings are dedicated to intradisciplinary centers, while interdisciplinary and comprehensive services take precedence over intradisciplinary services in rehabilitation centers.^24^ Furthermore, centers with interdisciplinary services provide high quality services and are highly cost-effective.^25^ Norrefalk et al evaluated multi-professional rehabilitation programs in musculoskeletal patients. They reported that the benefit of the program was estimated to be €3799–7515 per treatment.^26^ Cooney et al investigated the cost-benefit of an interdisciplinary rehabilitation team in patients with brain injury in the inpatient unit of a hospital. The results showed that as the patients’ quality of life increased, weekly care costs fell from €629 to €242.^27^

 It appears that the most significant reasons for not establishing outpatient rehabilitation centers include lack of insurance coverage for rehabilitation services and consequently high out-of-pocket expenses, the non-governmental sector’s unwillingness to establish rehabilitation centers due to the strict regulations, lack of special facilities to support the non-governmental sector in establishing and equipping rehabilitation centers, the policy limitations of the health care department concerning rehabilitation, lack of integrated and coordinated management of the meta-organization concerning rehabilitation, and neglect of rehabilitation services in the health care referral system that disrupts patients’ timely access to such services. The above reasons may also cause rehabilitation services not to be commensurate with the needs of people with disabilities in terms of quantity and quality.

 Moreover, the limited rehabilitation centers in Iran suffer from an improper distribution, causing unequal access to rehabilitation services, confusion in accessing such services, and an increase in the costs of such services. All these factors may reduce the effectiveness of rehabilitation services. Day care centers have been developed due to the subsidies of the Welfare Organization.

 Since services in rehabilitation centers are not covered by insurance, the private sector cannot generate acceptable income if it does not pay the allowance.

 In CBR, people with disabilities often need home rehabilitation after discharge from acute hospital care or outpatient rehabilitation programs to achieve rehabilitation goals and become reactivated in the community.^28^ Therefore, the WHO strongly recommends the development of CBR services.^29^

 In Canada, home rehabilitation services have been introduced since 1996 as a competitive model to offer high-quality, low-cost rehabilitation services that provide greater satisfaction and accessibility. However, the model has created major obstacles in achieving effective competition in this type of services. These obstacles include the limited number of private institutions willing to provide home rehabilitation services, the presence of structural barriers regarding teamwork and integration of services, and inadequate foundation for adding new providers. Studies have shown that if these barriers are not removed and these services are provided haphazardly and without supervision, their costs will not be reduced and their quality will not be improved. Limitations have been observed in this regard, which hinder reforms in the health system.^30^ A study conducted on stroke patients in 2017 showed that not only the medical and hospital costs of patients were reduced, but also the duration of patient care was shortened and their satisfaction improved.^31^ Another study performed at the London Institute of Aging and Rehabilitation in 2019 showed that CBR services had a net financial profit of more than $43 655 compared to routine care and were also more effective. The results also showed that the cost-effectiveness of this program was higher than conventional care in 100% of cases.^32^ There is much scientific evidence about the positive outcomes of home rehabilitation programs and CBR services in high-income countries.^33^ In Iran, CBR services are provided through home rehabilitation services (under the supervision of the State Welfare Organization), medical rehabilitation home care, and home rehabilitation nursing care (under the supervision of the Ministry of Health and Medical Education). According to the results of the present study, the number of rehabilitation centers and their distribution are not commensurate with the needs of the society.

 It appears that there are various obstacles in the country against developing rehabilitation centers, including policy makers’ insufficient knowledge about home rehabilitation and their insufficient attention to such issues as continuation of the treatment process, poor cooperation and coordination between rehabilitation centers, people with disabilities, and families regarding the benefit of rehabilitation services and management of their costs, high out-of-pocket payments, strict licensing requirements for the private sector, the limited ability of people with disabilities in using home rehabilitation services without subsidies or insurance, and the growth of unlicensed centers.

 Long-term care services are offered to maintain rehabilitation care in different settings. People visiting long-term rehabilitation centers usually have one or more chronic diseases or disabilities and need periodic, intermittent, or continuous care.^34^ In Iran, long-term rehabilitation care is provided to the elderly and people with disabilities in the form of residential care services. There is a greater number of long-term rehabilitation centers in the country compared to day-care and home rehabilitation centers. Thus, the State Welfare Organization in Iran aims to further develop day-care and home rehabilitation centers. This is because families who are unable to care for a disabled family member at home and do not have access to proper day-care and home care services are forced to pay the high cost of residential care centers. Therefore, the private sector is also more willing to invest in establishing residential care centers than day-care and home rehabilitation centers.

 Managers and stakeholders should be taught about health services with a rehabilitation and empowerment approach. Moreover, primary care training and its proper implementation should be monitored, which by itself can have a significant effect on reducing the consequences of disability and empowering people with disabilities.^35^

 A report on the availability of rehabilitation workforce showed that the density and distribution of rehabilitation workforce were inappropriate. However, the significant reason for the poor growth and development of rehabilitation workforce is the low and inappropriate development of rehabilitation centers (e.g., inpatient rehabilitation beds and outpatient rehabilitation centers). These centers provide the necessary space to use the existing capacity and a suitable platform for both teamwork and training to increase the rehabilitation team’s skills so that they can diagnose and perform interventions on complex diseases in a timely manner. Otherwise, rehabilitation services become specialty services with poor effectiveness and high costs. Currently, in Iran, the distribution of rehabilitation specialists and the establishment of rehabilitation centers and institutions have been more dependent on economic conditions and health care facilities and have not been commensurate with people’s needs for rehabilitation services. Due to the poor distribution of rehabilitation specialists and the insufficient development of rehabilitation centers in Iran, it appears that development policies should focus first on establishing more rehabilitation centers and then on increasing rehabilitation workforce. The WHO recommends that rehabilitation workforce and centers should focus on all the three levels, i.e. primary, secondary, and tertiary.^36^ However, according to the present study, a small number of people with disabilities have been able to receive the required services. The WHO’s Global Atlas of the Health Workforce^37^ shows that there is a shortage of qualified rehabilitation workforce worldwide, including Iran. As a result, rehabilitation needs have remained unfulfilled and thus the performance level will not improve in people, especially those in need of such services.^38,39^ Therefore, it appears that in all health systems around the world, especially in middle- and low-income countries, policies should be reformed and more rehabilitation facilities should be developed.
